# Western Cultural Identification Explains Variations in the Objectification Model for Eating Pathology Across Australian Caucasians and Asian Women

**DOI:** 10.3389/fpsyg.2016.01578

**Published:** 2016-10-14

**Authors:** Charmain S. Tan, Matthew Fuller-Tyszkiewicz, Ranjani Utpala, Victoria Wai Lan Yeung, Tara De Paoli, Stephen Loughan, Isabel Krug

**Affiliations:** ^1^Melbourne School of Psychological Sciences, The University of MelbourneMelbourne, VIC, Australia; ^2^Department of Psychology, National University of Singapore, SingaporeSingapore; ^3^Centre for Social and Early Emotional Development, School of Psychology, Deakin UniversityMelbourne, VIC, Australia; ^4^School of Psychology, Deakin University, DeakinMelbourne, VIC, Australia; ^5^Department of Applied Psychology, Lingnan University, Hong KongHong Kong; ^6^Department of Psychology, University of EdinburghEdinburgh, UK

**Keywords:** objectification, body shame, appearance anxiety thin-ideal internalization, eating pathology, cultural, western cultural identification

## Abstract

**Objective:** To assess differences in trait objectifying measures and eating pathology between Australian Caucasians and Asian women living in Australia and in Hong Kong with high and low levels of western cultural identification (WCI) and to see if exposure to objectifying images had an effect on state-objectification. A further aim was to assess using path analyses whether an extended version of the objectification model, including thin-ideal internalization, differed depending on the level of WCI.

**Method:** A total of 424 participants comprising 162 Australian Caucasians and 262 Asians (*n* = 183 currently residing in Australia and *n* = 79 living in Hong Kong) took part in the study. Of the overall Asian sample, 133 individuals were classified as high-WCI and 129 participants as low-WCI. Participants were randomly allocated into one of two conditions, presenting either objectifying images of attractive and thin Asian and Caucasian female models (objectification group, *n* = 204), or showing neutral images of objects (e.g., chairs, tables; control group, *n* = 220). Subsequently, participants were asked to complete a series of questionnaires assessing objectification processes and eating pathology.

**Results:** Findings revealed that the Caucasian group presented with significantly higher internalization and body surveillance scores than either of the two Asian groups and also revealed higher scores on trait-self-objectification than the low-WCI Asian sample. As regards to the effects of objectifying images on state self-objectification, we found that ratings were higher after exposure to women than to control objects for all groups. Finally, multi-group analyses revealed that our revised objectification model functioned equally across the Caucasian and the high-WCI Asian group, but differed between the Caucasian and the low-WCI Asian group.

**Conclusion:** Our findings outline that individuals with varying levels of WCI might respond differently to self-objectification processes. Levels of WCI should therefore be taken into consideration when working with women from different cultural backgrounds.

## Introduction

Objectification theory, developed by Fredrickson and Roberts (1997), proposes a formal framework that allows incorporation of both sociocultural (including media influences) and psychological risk factors, and their interactions with eating pathology. The literature has also advocated for the inclusion of internalization of the media ideal as preceding self-objectification, however, studies incorporating this variable have been scarce (Moradi and Huang, 2008). Most of the studies supporting the model have been correlational and only more recently have studies established growing support for the model using experimental designs (Harper and Tiggemann, 2008) or structural equation modeling (SEM) and/or path analyses (Tiggemann and Williams, 2012; Dakanalis et al., 2015a,b). Moreover, cross-cultural validation of the objectification model using these designs have been extremely limited (Kim et al., 2014), and the extent to which participants had identified with western ideals was not directly measured in the few cultural studies, even though it would likely impact the effects of ethnicity on objectification-related outcomes (Doris et al., 2015). The present study assessed, for the first time, within an Australian and Hong Kong context, differences across three groups differing in levels of western cultural identification (WCI) (Caucasians, high-WCI Asians and low-WCI Asians). It should be noted that the cultural norms in Australia and Hong Kong (given that it used to be a British colony), align with the thin ideal in other western cultures (Jennings et al., 2006; Lai et al., 2013); and that Australia has a high proportion of Asian heritage individuals. In specific the current study assessed differences across these three groups in trait objectification processes and eating pathology and used an experimental design to investigate whether exposure to objectifying media images had an effect on state-objectification in these three groups. Additionally, this study examined, using path analyses, whether a revised version of the objectification model, including thin-ideal internalization, varied depending on the level of WCI of the participants.

Objectification theory (Fredrickson and Roberts, 1997) asserts that women, through gender socialization and repeated experiences of sexual objectification (e.g., sexual harassment, exposure to media that objectify women), begin to take on an observer’s perspective of their body, and perceive and consider themselves as objects to be judged based on appearance (i.e., they self-objectify). Self-objectification is characterized by habitual monitoring of one’s outward appearance. The literature further distinguishes between state and trait self-objectification. The former refers to self-objectification that occurs as a consequence of an objectifying encounter or within a specific context (e.g., experimentally induced), while the latter relates to intrapersonal characteristics, which tends to be more stable, though in this instance, is still influenced by longstanding socialization processes (Moradi and Huang, 2008). Self-objectification is the primary component of the objectification model, and the mechanism by which exposure to a cultural environment that encourages objectification of women results in psychological problems.

**FIGURE 1:**
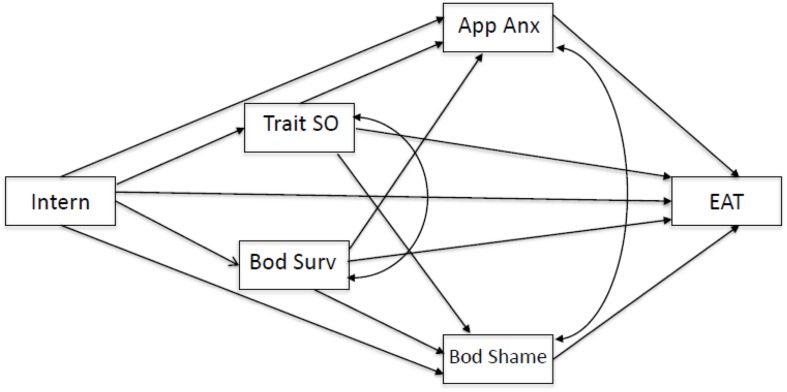
**A conceptual model of the revised objectification model including thin-ideal internalization.** Intern, internalization; App Anx, appearance anxiety; Bod Surv, body surveillance; Trait SO, trait self-objectification; Bod Shame, body shame; EAT, eating pathology.

More recently, the literature has advocated for the inclusion of internalization as preceding self-objectification (Moradi and Huang, 2008; Tiggemann, 2013; Dakanalis et al., 2014). Internalization of the media ideal refers to the extent an individual endorses and engages in behaviors which helps them to abide by societal archetypes of attractiveness (Harrison and Hefner, 2006). While most women in westernized countries are exposed to the pervasive thin-ideal female form and the pressure to conform, not all of them go on to experience adverse psychological outcomes. It has been argued that the adoption of cultural standards of beauty (i.e., internalization of the thin ideal) is a key mediating variable between exposure to sexual objectification and self-objectification, psychological issues and maladaptive eating patterns, and should therefore be included in future objectification studies (Moradi and Huang, 2008). **Figure 1** outlines the objectification model, including thin-ideal internalization, as it relates to eating pathology.

Correlational studies of the objectification model (with or without internalization) have found relationships between self-objectification processes and levels of eating disorder symptoms across a variety of population types, including adolescents (Slater and Tiggemann, 2010), young females (Dakanalis et al., 2013, 2016), older woman (Augustus-Horvath and Tylka, 2009), physically active women (Greenleaf and McGreer, 2006), women with eating disorders (Calogero et al., 2005), deaf women (Moradi and Rottenstein, 2007), lesbian women (Kozee and Tylka, 2006) and heterosexual and gay men (Engeln-Maddox et al., 2011; Dakanalis et al., 2012).

In the few comparative studies between Caucasian and Asian women, Frederick et al. (2006, 2007), reported equal levels of body surveillance, but greater body dissatisfaction in Asian compared to Caucasian women, once body mass index (BMI) was statistically controlled. However, despite differences in body dissatisfaction, there was a similar body surveillance-body dissatisfaction relationship across both groups. Another study comparing Caucasian and Asian women revealed that self-objectification was related to body shame and surveillance in both groups, despite higher trait self-objectification, body surveillance and body shame scores in the Caucasian group (Claudat et al., 2012). Therefore, these studies suggest that the relationships conceived within the objectification model might be similarly applicable to Asian women, however, further experimental studies and research using more advanced statistical procedures such as SEM and/or path modeling in cultural diverse samples are required to verify these initial findings.

To date, experimental studies that have tested the effect of objectifying media images on state self-objectification have been relatively scarce. One of the key studies in this area, elicited state self-objectification by exposing women to advertisements taken from fashion magazines marketed toward young adult women (Harper and Tiggemann, 2008). Those in the control condition were shown images featuring products without people, while those in the two experimental groups were shown images containing thin women with or without attractive men and four images from the control condition. The researchers found that women from the experimental groups had greater state self-objectification, weight related appearance anxiety, negative mood states, and body dissatisfaction compared to individuals who viewed the control images. However, no differences were identified across conditions for non-weight-related appearance anxiety. Aubrey et al. (2009) similarly investigated the impact of showing images of female models with high skin exposure, women’s body parts or models with low skin exposure on self-objectification and criterion variables. They found that participants in the high skin exposure condition used more negative words to describe their appearances and had greater state self-objectification relative to the other conditions. To our knowledge there is no study that has assessed the effect of media images on state self-objectification in a culturally diverse sample. Further cross-cultural experimental research in this area is therefore required.

Recent studies have incorporated SEM and/or path analyses in their analyses, enabling simultaneous assessment of all of the relationships within the objectification model (Tylka and Sabik, 2010; Calogero and Pina, 2011; Tiggemann and Williams, 2012; Dakanalis et al., 2015a,b). Most of these studies have found support for the pathways within the objectification model. In a comprehensive test of the objectification model using SEM, Tiggemann and Williams (2012), found a sufficient fit of the model within a primarily white female population. Significantly, the model accounted for 93% of the variance in predicting eating disorder symptoms, with both body shame and appearance anxiety as major mediators. Additionally, past studies have also used SEM to demonstrate the role of internalization in contributing to body image disturbances and subsequently, eating pathology (Tylka and Subich, 2004; Moradi et al., 2005; Kozee and Tylka, 2006). However, there appears to be only one study exploring the applicability of the objectification model in an Asian population (Kim et al., 2014) living in their home country. In university age Asian-born South Korean women, internalization, body surveillance, and body shame were found to mediate the relationship between media exposure and maladaptive eating (Kim et al., 2014). This provides support for the generalizability of the objectification model to a South Korean cohort, and raises the possibility that the model could be extended to other Asian populations as well. However, a limitation of that study was that the sample assessed might have varied considerable as regards to having adapted to the western cultural ideal, which could have been accounted for by assessing the level of acculturation to the western culture.

It is possible that Asian women experience changes in terms of their body image and eating behaviors when they start identifying more with the Western cultural norms. Thus far, research on the links of WCI with body image and eating disorder symptoms has produced mixed findings, with a recent systematic review (Doris et al., 2015) on the topic of acculturation, outlining that both higher and lower acculturation levels have been identified as risk factors for the development of eating disorders in Asian women (Doris et al., 2015). The same review also outlined that these inconsistent findings could partially be explained by the different acculturation measures employed in the studies reviewed. Further research on WCI, in relation to the objectification model in cross-cultural studies using validated measures assessing various components of acculturation and WCI (e.g., language, identity, friendships, behaviors, generation background and attitudes) are therefore needed (Suinn et al., 1992).

To conclude, existing research examining objectification theory tenets has largely used convenience samples of white, upper middle class, undergraduate women. Thus, there continues to be limited experimental research and SEM and/or path analyses studies in ethnically and culturally diverse sample. Research has also highlighted the relationship between internalization and various factors within the objectification model. However, despite this association, the inclusion of internalization in objectification theory research continues to be limited across SEM and/or path analysis studies. Finally, no study to date has assessed the role of WCI on the model when assessing individuals from different cultural and ethnic backgrounds. However, including WCI into the model is important, as it might explain differences between the objectification theory constructs and/or relationships, further increasing our understanding of the model across cultures. Consequently, it may also allow for more efficacious preventive measures and interventions techniques to reduce the risk of eating disorders in other cultures.

The present study aimed to integrate culture into the objectification framework for eating pathology among Caucasian Australians and Asians with high and low levels of WCI. More specifically, we aimed to partially replicate Harper and Tiggemann’s (2008) research by assessing the effect of objectifying images (thin-ideal Caucasian and Asian women), compared to neutral pictures (chairs, tables), on ratings of state-self objectification and to assess what impact WCI played in both state and trait objectification processes and eating pathology. Hence, our aims were threefold: (1) to assess differences across trait-level variables (appearance anxiety, body shame, surveillance, trait self-objectification and eating pathology), across three groups including Caucasians and Asians with either high or low levels of WCI; (2) to examine the effect of objectifying images on state self-objectification across these three groups; and (3) to undertake multi-group path modeling to explore whether a (revised) objectification model, including thin-ideal internalization, differed across the three groups (see **Figure 1**). Internalization was added to the model since it would further aid us in understanding the objectification model and it would also provide clarification of posited WCI group differences.

## Materials and Methods

### Sample

The sample comprised 424 Asian and Caucasian women between the ages of 17–48 years, (*M* = 19.72, *SD* = 3.72), who were recruited from a university in Australia (*n* = 345) and a university in Hong Kong (*n* = 79). Study eligibility criteria included being female, and of either Asian or Caucasian descent. Of the participants, 38.2% (*n* = 162) were Australian Caucasian, 20.8% (*n* = 88) were Australian Asian, 22.4% (*n* = 95) were Asian who had moved to Australia for educational purposes, and 18.6% (*n* = 79) were Asian women living in Hong Kong. The mean body mass index of the overall sample was 21.15 kg/m^2^ (*SD* = 3.21). Ethical approval was obtained from a University in Melbourne and a University in Hong Kong.

### Design

An experimental between-subjects design was used to assess the impact of objectifying images on the variables in the objectification theory. Participants were randomly allocated into one of two conditions, one presenting 40 objectifying images of both attractive and thin Asian and Caucasian female models (objectification group), while the other were shown 40 neutral images of objects (control group). A little bit less than half of the total sample (*n* = 204) were in the objectification group while the other participants (*n* = 220) were in the control group.

### Choice of Images

The images of women were generated with an online search using descriptors related to sexual objectification (e.g., “attractive,” “thin,” “objectification,” “sexy,” “Asian”). Each picture chosen portrayed a thin-ideal woman, who looked either Asian or Caucasian, in sexualized and/or objectified manners (e.g., postures which emphasized body parts, clothed in revealing garments, poses which portrayed sexual desire or interest). Attempts were made to avoid images of celebrities, which might result in greater attention or other inadvertent biases not present within the other stimulus. The initial pool of images was reduced to 20 Asian women and 20 Caucasian women by a group of female volunteers (*N* = 10). They were asked to rate how attractive the images within each set were using a five-point scale (1 = Unattractive; 5 = Attractive). Images with the highest ratings were chosen for use in the experiment. The pictures of objects were selected using a similar process, changing only the keywords used (e.g., “accessories,” “home ware”).

### Measures

#### Demographics

Information on age, country of birth, years lived in Australia, ethnicity, weight, and height were obtained. BMI was subsequently calculated as the ratio of weight (kg) to height squared (m^2^).

#### State Self-Objectification

A shortened version of the Twenty Statements Test (TST; Fredrickson et al., 1998) was used to assess experimentally heightened changes in self-objectification. Participants were asked to describe themselves by completing ten sentences about their identity that begin with the phrase “I am.” Reponses were coded by two raters (C.T and T.P.) and categorised into six categories: (a) body shape and size, (b) other physical appearance, (c) physical competence, (d) traits or abilities, (e) states or emotions and (f) uncodable or illegible. Scores were derived by summing up the number of responses from the two appearance-related categories (i.e., a and b). Scores ranged from 0 to 10 with higher scores indicative of greater state self-objectification. The second coder (T.P.) coded a random sample of the statements, with a 97.6% agreement to the original ratings as to the responses either being appearance-or not appearance-based.

#### Trait Self-Objectification

There are two common methods of operationalizing trait self-objectification within the literature. One approach is through the Body Surveillance subscale from the Objectified Body Consciousness Scale (OBCS; McKinley and Hyde, 1996), which is behavioral in nature as it assesses level of reported habitual body monitoring. The other is via the Self Objectification Questionnaire (SOQ; Noll and Fredrickson, 1998), which looks at the cognitive component of self-objectification, comparing participants’ perceived importance of appearance- versus competence-based body attributes. Currently, it remains unclear if the Body Surveillance subscale and SOQ assess distinct or overlapping or similar construct(s). As such, researchers (Moradi and Huang, 2008) have argued for the use of both to address the process of self-objectification more comprehensively.

##### Body surveillance

The Body Surveillance subscale, taken from the OBCS (McKinley and Hyde, 1996), evaluates the extent to which individuals monitor their bodies as an observer and think about their bodies in terms of how it looks. There are eight items (e.g., *“I rarely compare how I look with how other people look”*), which were rated on a scale ranging from 1 (*Strongly Disagree*) to 7 (*Strongly Agree*). Scores were averaged, with higher scores illustrating more time expended on self-monitoring and greater concern for outward appearances. In previous studies, Cronbach’s alphas have ranged from 0.76 to 0.89 with a test–retest reliability of.79 (McKinley and Hyde, 1996). The internal consistency in this study was 0.82.

##### Trait self-objectification

The SOQ (Noll and Fredrickson, 1998) assesses the extent to which participants have a primarily appearance-based versus competence-based self-concept. In the current study, participants were asked to rank 10 attributes in order of how important the parts were to their self-concept (with 1 being *most important* and 10 being *least important*). Scores were obtained by summation of the ranks within the appearance and competency items, and computing the difference of appearance from competency. Scores ranged from -25 to 25, with higher scores indicative of the greater importance of appearance, which was interpreted as higher trait self-objectification.

#### Body Shame

The Body Shame subscale from the OBCS (McKinley and Hyde, 1996) assesses the level of guilt an individual experiences for not attaining the cultural standard (e.g., “*I feel ashamed of myself when I haven’t made the effort to look my best*”). It consists of 8 items, with ratings based on a 7-point scale ranging from 1 (*Strongly Disagree*) to 7 (*Strongly Agree*). Scores were derived by averaging the responses given, with higher scores indicative of greater body shame. In a previous study, a 2-week test–retest reliability of 0.79 and internal consistencies ranging from 0.70 to 0.84 were reported (McKinley and Hyde, 1996). Cronbach’s alpha in the current sample was 0.81.

#### Appearance Anxiety

This was measured using the brief version of the Appearance Anxiety Scale (Dion et al., 1990), which assesses preoccupation with observable aspects of the physical self and body image assessment. Respondents reported the extent to which each of 14 statements (e.g., “*I get nervous when others comment on my appearance*”) were true of them (0 = *never*; 4 = almost *always*). Scores were achieved by summation, with higher scores demonstrating higher anxiety about one’s appearance. A previous study reported the internal consistency to be 0.86, with a test-retest reliability of 0.89 (Dion et al., 1990). In the present study, the scale had an internal consistency of 0.90.

#### Internalization of Media Ideals

The General-Internalization subscale from the Sociocultural Attitudes Toward Appearance Scale-3 (SATAQ-3; Thompson et al., 2004) was administered to assess the internalization and acceptance of societal pressure to be thin and attractive. It consists of nine items (e.g., *“I compare my body to the bodies of people who are on TV”)* and participants respond on a scale ranging from 1 (*completely disagree*) to 5 (*completely agree*). Scores were obtained by averaging item responses, with higher scores representative of greater internalization. A previous study reported Cronbach’s alpha of 0.92 (Thompson et al., 2004). Within the current sample, it had an internal consistency of 0.93.

#### Eating Pathology

The Eating Attitudes Test (EAT-26; Garner et al., 1982) was used to determine the level of disordered eating attitudes and behaviors in participants. It is a widely used screening tool consisting of 26-items on a 6-point scale ranging from *Always* to *Never*. These items were designed to measure level of dieting, bulimia and food preoccupation, as well as oral control. Dieting was measured by 13 items (e.g., “*Am preoccupied with a desire to be thinner*”); bulimia and food preoccupation by six items (e.g., “*Have gone on eating binges where I feel that I may not be able to stop*”); and oral control by seven items “*Display self-control around food.*” The EAT-26 has excellent psychometric properties with reliability coefficients ranging between 0.70 and 0.88 (Garner et al., 1982). Cronbach’s alpha for the overall EAT-26 scores in the current sample was 0.86.

#### Western Cultural Identification

The Suinn-Lew Asian Self-Identity Acculturation Scale (Suinn et al., 1992) is a 21-item scale that assesses different levels of acculturation, including language, identity, friendships, behaviors, generation background and attitudes. A higher composite score reflects greater western identification, whereas a lower composite score is indicative of greater identification with Asian culture (i.e., low-WCI). To examine if and how levels of WCI in Asian participants impacted the objectification model, Asian respondents were categorized into two groups (high- and low-WCI), with 133 participants in the former and 129 in the latter. This was achieved by conducting a median-split on the composite score of the acculturation scale. Reliability of the scale was reported as ranging from 0.88 to 0.91 (Suinn et al., 1992). Cronbach’s alpha in this study was 0.97.

### Procedure

The study was administered online via Qualtrics. At the start of the study, participants read and signed a consent form informing them of the voluntary nature of the study, before completing the demographic questions. Next, participants were briefed that they would be viewing a slideshow of 40 images, however, participants were blind to the purpose of the study. Allocation into the condition was randomized by Qualtrics, based on when they began the study and self-selected ethnicity (Asian or Caucasian). The latter was done to ensure roughly equal representation of both groups across the experimental and control conditions. Participants were encouraged to attend to the images, and informed that they would be asked about them later in the study. Forty pictures of either objectified women or control were shown, one at a time, for 3 s each. Once the slideshow was completed, a battery of questionnaires assessing the variables in the objectification framework, eating pathology and WCI was administered.

### Statistical Analyses

All descriptive and group-difference based analyses were conducted using IBM SPSS 20.0, whereas path analyses were conducted in Mplus. Between group analyses (using *t*-test, chi-square, and one-way ANOVAs, as appropriate) of the sociodemographic factors were done to identify differences between women across WCI levels (Caucasian versus high-WCI Asian versus low-WCI Asian participants). MANOVA analyses were used to assess the impact of WCI level on trait self-objectification, internalization, body surveillance, body shame, appearance anxiety, and maladaptive eating behaviors. A 2 × 3 ANOVA was undertaken to evaluate the moderating effect of WCI levels on the relationship between images viewed (objectifying versus control) and state self-objectification.

Finally, a series of multi group path analyses was undertaken to test the model shown in **Figure 1**. As we were interested in the possibility that the Caucasian group differed from the other two groups, separate analyses were undertaken to compare Caucasian versus low-WCI Asian participants and Caucasian versus high-WCI Asian participants. In both of these comparisons, model parameters were set to be equal across groups (e.g., the relationship between internalization and appearance anxiety was forced to be of equal magnitude for the Caucasian and the low-WCI groups), and model fit was compared against a saturated model (since the model with parameters freely estimated across groups consumed all degrees of freedom).

Standard cut-offs were used to evaluate acceptable model fit for the model in which parameters were constrained to equality: non-significant chi-square value, Comparative Fit Index (CFI) >0.95, Root Mean Square Error of Approximation (RMSEA) <0.06, and Standardized Root Mean Square Residual (SRMR) <0.08 (Hu and Bentler, 1999; Byrne, 2012). As the model in which parameters were allowed to vary across groups was saturated (i.e., χ^2^ = 0, df = 0, CFI = 1, RMSEA = 0, and SRMR = 0), fit statistics are not reported for this model. As a consequence, the models were concluded to be different across groups if change in chi-square (i.e., chi-square for the constrained model minus chi-square for the baseline model) was significantly different from zero (see Byrne, 2012 for examples using invariance testing within the Mplus framework). In this instance, as the baseline (unconstrained) model is saturated, the change in chi-square statistic and its significance is identical to those reported for the constrained model. Standardized coefficients are reported in-text. Studies have identified age and BMI as potential covariates of eating disorder constructs (Augustus-Horvath and Tylka, 2009). In order to provide a more stringent test of the hypotheses, age and BMI were therefore added as covariates for all other variables in the path models.

## Results

### Sociodemographics

The sociodemographic variables for the overall sample, Caucasian, high-WCI Asian, and low-WCI Asian groups are presented in **Table 1**. Significant group differences were observed for BMI (*p* < 0.001), but not for age (*p* = 0.104). The low-WCI Asian (*p* < 0.001) and the high-WCI Asian groups (*p* = 0.001) had lower BMI compared to Caucasian women. There were no significant differences between the two Asian groups (*p* = 0.597) for BMI. Distribution of participants across the education level categories (secondary, tertiary, and postgraduate) differed significantly between the three WCI groups (*p* < 0.001), with both of the Asian groups tending to have a higher proportion of participants completing tertiary studies than the Caucasian group, and this proportion was greater for the low-WCI Asian group than the high-WCI Asian group.

**Table 1:** Sociodemographic details of study participants.

	Total (*n* = 424)	Caucasian (*n* = 162)	High-WCI Asian (*n* = 133)	Low-WCI Asian (*n* = 129)
**N (%)**				
University student	418 (98.58)	162 (100)	132 (99.25)	124 (96.12)
**Highest completed education:**				
Secondary	290 (68.40)	133 (82.10)	97 (72.93)	60 (46.51)
Tertiary	123 (29.01)	26 (16.05)	34 (25.56)	63 (48.84)
Postgraduate	11 (2.59)	3 (1.85)	2 (1.50)	6 (4.65)
**Mean (*SD*)**				
Age (years)	19.71 (3.72)	20.06 (4.77)	19.16 (3.02)	19.87 (2.68)
BMI	21.15 (3.21)	22.06 (3.24)	20.40 (3.25)	20.77 (2.88)

*WCI, western cultural identification.*

### The Impact of WCI on Trait Measures from the Objectification Theory

**Table 2** provides a breakdown by group (Caucasians, high-WCI and low-WCI Asians) of means for each of the trait variables from the objectification theory. Across most variables, Caucasian participants reported higher levels for each of the study variables, followed by the high-WCI Asian group, and then the low-WCI Asian group. A series of ANOVAs was conducted to evaluate WCI-related differences in the trait variables from the objectification theory. As detailed in **Table 2**, significant univariate effects were observed for: (1) trait self-objectification – the low-WCI Asian group had significantly lower levels of objectification than the Caucasian group; (2) body surveillance – the Caucasian group reported higher levels of body surveillance than either of the Asian subgroups, and the high-WCI Asian group had higher levels of body surveillance than the low-WCI Asian group; and (3) internalization – the Caucasian group reported higher levels of internalization than either of the Asian subgroups. Group differences for body shame, appearance anxiety, and eating pathology were all non-significant, and thus, pairwise comparisons were not undertaken for these outcome measures.

**Table 2:** Descriptive statistics and group difference tests for trait measures.

	Caucasians (*n* = 162)	High-WCI Asian (*n* = 133)	Low-WCI Asian (*n* = 129)	*F*(2,421)	*p*	η^2^
Trait self-objectification	1.10 (12.42)^a^	-1.26 (14.34)	-4.12 (12.05)^c^	5.83	0.003	0.027
Body surveillance	4.87 (0.96)^a,b^	4.55 (1.03)^a,c^	4.31 (0.84)^b,c^	12.70	<0.001	0.057
Body shame	3.58 (1.17)	3.38 (1.08)	3.62 (0.88)	2.29	0.130	0.010
Appearance anxiety	31.31 (10.34)	29.64 (9.47)	29.93 (6.95)	1.43	0.241	0.007
Internalization	3.46 (0.94)^a,b^	3.16 (0.94)^c^	3.01 (0.85)^c^	9.15	0.000	0.042
Eating pathology	10.78 (10.98)	10.95 (9.31)	9.03 (7.71)	1.65	0.193	0.008

*WCI, western cultural identification. ^a^Group differed significantly (p < 0.05, two-tailed) from less acculturated group, ^b^Group differed significantly from more acculturated group, ^c^Group differed significantly from Caucasian group.*

### The Impact of Image Type and WCI on State Self-Objectification

A 2 × 3 factorial ANOVA was conducted using image type (objectification, control) and level of WCI (Caucasian, high-WCI Asian and low-WCI Asian) as the independent variables, and state self-objectification as the dependent variable. There was no significant interaction effect, *F*(2,418) = 1.30, *p* = 0.273, $\eta_{p}^{2}$ = 0.006. A significant main effect of image type, *F*(1,418) = 10.64, *p* = 0.001, $\eta_{p}^{2}$ = 0.025 was present, with higher state self-objectification for images of women (*M* = 1.01, *SD* = 1.16) than for objects (*M* = 0.65, *SD* = 0.99). The main effect for WCI was borderline significant, *F*(2,418) = 3.43, *p* = 0.051, $\eta_{p}^{2}$ = 0.022, and thus *post hoc* comparisons were undertaken. These pairwise comparisons showed that the low-WCI Asian group had significantly higher state self-objectification (*M* = 1.02, *SD* = 1.11) than the Caucasian group (*M* = 0.73, *SD* = 1.03; *p* = 0.021), but the high-WCI Asian group (*M* = 0.76, *SD* = 1.11) did not significantly differ from the low-WCI Asian group (*p* = 0.060) or the Caucasian group (*p* = 0.736).

### Comparing the Revised Objectification Theory Model Across the WCI Groups

Multi-group path analyses showed that the proposed, objectification theory model functioned equivalently across Caucasian and the high-WCI Asian groups [χ^2^(*df* = 25) = 27.73, *p* = 0.320; CFI = 0.996, RMSEA = 0.027, SRMR = 0.058], but differed for Caucasian and the low-WCI Asian groups [χ^2^(*df* = 25) = 64.92, *p* < 0.001]. Unsurprisingly, fit for the model in which Caucasian and the high-WCI Asian groups were constrained to equality was acceptable (CFI = 0.996, RMSEA = 0.027, SRMR = 0.058), but poor for the model constraining Caucasian and the low-WCI Asian groups to equality (CFI = 0.939, RMSEA = 0.105, SRMR = 0.148). Given this pattern of results, model parameters are reported separately for: (1) the Caucasian and high-WCI Asian group combined and (2) the low-WCI Asian group.

#### Caucasian/High-WCI Asian Group

**FIGURE 2:**
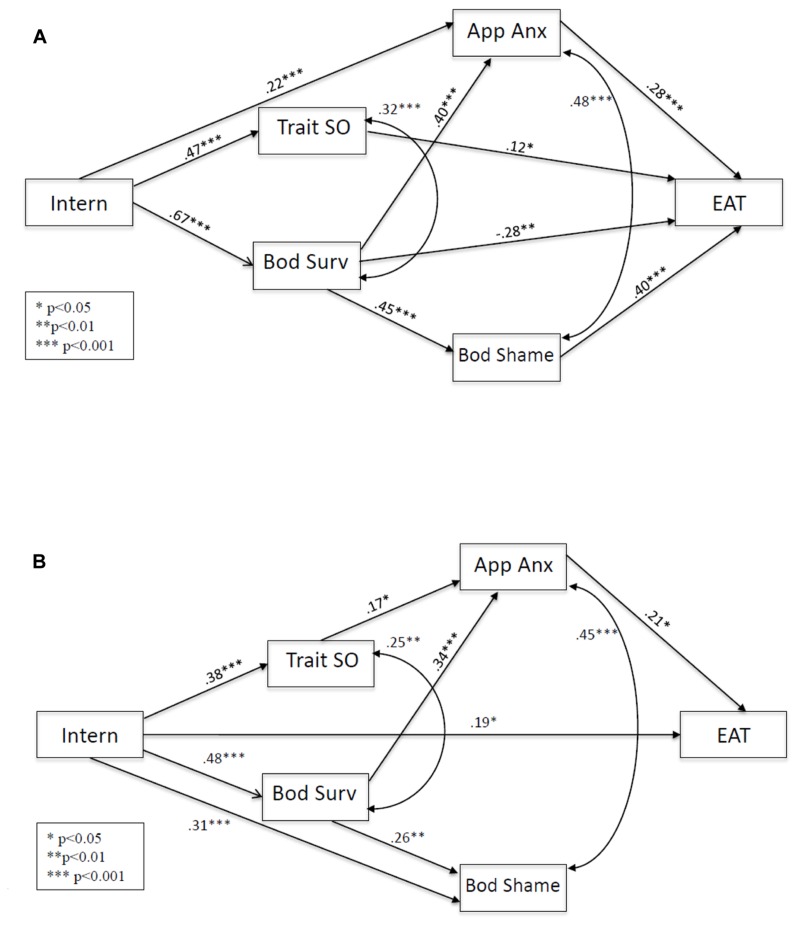
**Differences in the revised objectification model across the WCI groups. (A)** Significant pathways for the Caucasian/High-WCI Asian group. **(B)** Significant pathways for the low-WCI Asian group. Covariates (age and BMI) and non-significant pathways are omitted from the figure for clarity of presentation. Intern, internalization; App Anx, appearance anxiety; Bod Surv, body surveillance; Trait SO, trait self-objectification; Bod Shame, body shame; EAT, eating pathology.

As shown in **Figure 2A**, for the Caucasian/High-WCI Asian group model, eating pathology was significantly predicted by appearance anxiety, body shame, body surveillance, and trait self-objectification. Appearance anxiety was significantly predicted by body surveillance and internalization. Body shame was significantly predicted by body surveillance. Trait self-objectification and body surveillance were both predicted by internalization. Appearance anxiety co-varied with body shame, and trait self-objectification co-varied with body surveillance.

Internalization had several significant indirect effects on eating pathology, body shame, and appearance anxiety: (1) internalization → body surveillance → eating pathology, β = -0.19, *p* = 0.002; (2) internalization → body surveillance → body shame → eating pathology, β = 0.12, *p* < 0.001; (3) internalization → body surveillance → appearance anxiety → eating pathology, β = 0.08, *p* = 0.002; (4) internalization → appearance anxiety → eating pathology, β = 0.06, *p* = 0.011; (5) internalization → trait self-objectification → eating pathology, β = 0.06, *p* = 0.041; (6) internalization → body surveillance → appearance anxiety, β = 0.27, *p* < 0.001; and (7) internalization → body surveillance → body shame, β = 0.30, *p* < 0.001.

In total, 34% of the variance in eating pathology (32% when covariates are excluded), 48% of the variance in appearance anxiety (41% when covariates are excluded), 46% of the variance in body surveillance (45% when covariates are excluded), 34% of the variance in body shame (29% when covariates are excluded), and 22% of the variance in trait self-objectification were accounted for by predictor variables (including the covariates) in the model (22% when covariates are excluded).

#### Low- WCI Asian Group

For the low-WCI Asian group (see **Figure 2B**), eating pathology was significantly predicted by appearance anxiety, and internalization. Appearance anxiety was significantly predicted by trait self-objectification and body surveillance. Body shame was significantly predicted by body and internalization. Trait self-objectification and body surveillance were both predicted by internalization. Appearance anxiety co-varied with body shame, and trait self-objectification co-varied with body surveillance.

Internalization had several significant indirect effects on body shame and appearance anxiety, but not for eating pathology: (1) internalization → trait self-objectification → appearance anxiety, β = 0.07, *p* = 0.029; (2) internalization → body surveillance → appearance anxiety, β = 0.16, *p* = 0.001; and (3) internalization → body surveillance → body shame, β = 0.12, *p* = 0.006.

In total, 25% of the variance in eating pathology (22% when covariates are excluded), 34% of the variance in appearance anxiety (28% when covariates are excluded), 22% of the variance in body surveillance (21% when covariates are excluded), 29% of the variance in body shame (25% when covariates are excluded), and 14% of the variance in trait self-objectification were accounted for by predictor variables (including the covariates) in the model (12% when covariates are excluded).

## Discussion

Our findings revealed significant differences in trait objectification measures across the three WCI groups, with the Caucasian group presenting with significantly higher internalization and body surveillance scores than the low and high WCI Asian groups and the Caucasian group also revealed higher scores on trait self-objectification than the low-WCI Asian sample. As regards to the experimental component of the study, we found higher scores in the group that viewed the thin-ideal images of women compared to the control group in all three groups, indicating that our exposure was successful in eliciting state self-objectification, but that this effect generalized across cultural groups. Finally, our revised objectification model, including thin-ideal internalization was equivalent across the Caucasian and high-WCI Asian group, but differed for the Caucasian and the low-WCI Asian individuals. For this reason, we combined our Caucasian and high-WCI Asian groups in the path analyses and compared this combined group to the low-WCI Asian group. Each of these findings will be discussed in more detail in the subsequent sections.

### Main Effect of WCI on the Assessed Trait Variables

Greater trait self-objectification, internalization and body surveillance were demonstrated in Caucasian women compared to Asian women, particularly the low-WCI Asian group. This is in line with previous findings, which have found that Asian American women had less internalization, lower trait self-objectification levels and lower body surveillance compared to Caucasian Americans (e.g., Claudat et al., 2012; McKenney and Bigler, 2016).

A further important finding was that there were no statistically significant differences in disordered eating scores across the different WCI groups. This finding is consistent with previous findings, in which Asian American (Marques et al., 2011; Nouri et al., 2011), and Australian women (Jennings et al., 2006) demonstrated comparable levels of weight concerns, the use of unhealthy weight control behaviors and eating pathology compared to Caucasian women, but contradicts the findings of other studies, which found higher eating pathology in Asian females (Jennings et al., 2005) compared to Caucasian Australians. Future studies are therefore required to disentangle these contradictory findings.

### Impact of Exposure to Media Images and WCI on State-Self-Objectification

As regards to the experimental aspect of the current study, we found that state self-objectification was higher in the group viewing objectifying images compared to the control group, suggesting that objectifying images were effective in inducing self-objectification. This finding is consistent with the findings of other studies (Harper and Tiggemann, 2008; Aubrey et al., 2009), which also found greater state self-objectification, weight related appearance anxiety, negative mood states, and body dissatisfaction, in response to viewing objectifying images. However, it should be noted that we were only able to assess state self-objectification in this experimental part of the study, given that the other measures were all trait-based. Future studies should include a range of state-related objectification and disordered eating measures to extend our findings.

Even though a significant interaction between the exposure group (objectifying versus control) and the level of WCI (Caucasian, high-WCI and low-WCI Asian groups) was not revealed, we found that the low-WCI Asian group exhibited the highest state self-objectification ratings after exposure to images of women. This finding could be attributable to the fact that perhaps the low-WCI Asian group had fewer opportunities to be exposed to the Western thin ideal, resulting in a more detrimental influence of these images in this group.

### Comparing the Revised Objectification Theory Model Across the WCI Groups

Despite some differences between the aforementioned variables across levels of WCI, invariance testing indicated that the objectification model was largely equivalent between Caucasians and high-WCI Asian women, but differed significantly between the Caucasians and the low-WCI Asian group. For this reason, a combined Caucasian/High-WCI Asian group was compared to a low-WCI Asian sample in the path analyses. Consistent with previous studies (e.g., Calogero and Pina, 2011; Tiggemann and Williams, 2012; Kim et al., 2014), this suggests that the objectification model may be used to explain and understand maladaptive eating development for high-WCI Asian women. Furthermore, these results are congruent with the literature, which highlights similar risk factors in the development of eating disorders between high-WCI Asian and Caucasian females (Pike and Dunne, 2015).

A closer look at the revised model also highlighted several areas of interest that may add to the understanding of the objectification theory in groups differing in level of WCI. First, the variance explained in these outcome measures tended to be lower for the low-WCI group, suggesting that in addition to differences in mean levels for these objectification variables, their associations may also differ across cultures. Second, the indirect effects of internalization on key outcomes (body shame, appearance anxiety and eating pathology) differed across the Caucasian/High-WCI and the low-WCI Asian groups. The key indirect effects can be summarized as follows: (1**)** body surveillance mediated the internalization-appearance anxiety and internalization-body shame relationships in both groups; (2) the internalization-eating pathology relationship was not mediated by any variables for the low-WCI Asian group, but was mediated by several variables for the Caucasian/High-WCI Asian group (body shame, trait self-objectification appearance anxiety and body surveillance, however, the relationship for this last variable was negative for the body surveillance-eating pathology pathway).

The mediating role of body shame and appearance anxiety in the links between internalization of cultural standards of beauty and body surveillance with eating pathology has been supported with young and adult women (e.g., Calogero et al., 2010; Rolnik et al., 2010; Dakanalis et al., 2015a). Furthermore, the finding that internalization acts as an antecedent to the objectification model, is congruent with previous research that highlighted both the direct and indirect unique contribution of internalization to the various self-objectification measures and eating pathology (Calogero et al., 2005; Moradi et al., 2005; Sinclair, 2005; Myers and Crowther, 2007; Dakanalis et al., 2015b).

Conversely, for the low-WCI Asian group, few significant pathways were revealed for the overall model, with no significant indirect pathway being found from internalization to eating pathology. It is possible that the low-WCI Asian group might have had certain protective factors, which prevented them from experiencing the negative psychological consequences of self-objectification, for instance, that these women were enculturated to their heritage culture (Sussman et al., 2007; Pike and Dunne, 2015) or they might have adopted an integration style of adopting to the WCI, accepting the identity of both cultures and therefore experienced less stress and subsequently less exposure to risk factors for eating pathology (Doris et al., 2015). Finally, it is also possible that the traditional Asian conceptualization of ideal female beauty emphasizes other body parts, as for example the face, rather than the body. Accordingly, Kim et al. (2014) found that face size and shape was one of the most important factors in the modified objectification model, tested in a South-Korean sample. Furthermore, research has shown that amongst Asians, there is relative homogeneity in terms of BMI variance (Bélanger et al., 2010) and therefore it is likely that these women may have developed a stronger preference to differentiate each other through other body parts such as for instance facial features. Further research is required to further assess all of these potential explanations.

Finally, it is also worth mentioning that the thin ideal internalization variable had a direct effect on eating pathology only in the low-WCI Asian group, but not the Caucasian/High-WCI Asian group. This finding is in agreement, with previous findings, outlining that the adoption of western cultural standards of beauty, in the form of the thin ideal internalization, has direct detrimental effects on eating pathology, especially in individuals that have previously been less exposed to the western culture (Stice, 2002; Dakanalis et al., 2013, 2016).

### Limitations

The present findings should be considered in light of a number of study limitations. A limitation of the current study is the reliance on undergraduate participants, which is a limiting characteristic of most experimental research in this field. The cross-sectional and correlational nature of the data does not allow for strong causal inferences from the study’s results. Furthermore, participants filled in the questionnaire-based measures after having seen the objectifying or neutral images to replicate the original experimental studies in this area (Harper and Tiggemann, 2008; Aubrey et al., 2009). This ordering of manipulation and measurement could have had an effect on the participants’ responses. A better design would have entailed a pre-post design, but given the extensive number of measures included in the current study this would have been too burdensome for the participants. We did, however, carefully consider our measures and made sure that with the exception of state self-objectification, all other measures were trait based. Hence, it seems unlikely that the trait measures might have been impacted by the images previously seen. Another consideration is that our Asian group was used as a homogenous sample in this study, despite being made up of various ethnic subgroups from various countries (e.g., Hong Kong, China, Singapore, Malaysia, Indonesia, and Korea). Although this adds breadth, important group differences might have been overlooked. It should also be considered that the group from Hong Kong was not living in Australia, however, given that Hong Kong used to be a British colony we assumed that individuals there would be exposed to a similar Western ideal as in Australia. It should also be noted that the path analysis approach we used, assumed that the constructs included in the assessed model functioned equally across the Caucasian and the two Asian groups. Future studies would benefit from using a multi-group Confirmatory Factor Analysis (CFA) framework, to explicitly test such an assumption. Finally, given the links established in prior studies, the proposed baseline model tested in the present study was saturated (i.e., all degrees of freedom were consumed). This is not the standard approach for group comparisons (where the baseline is typically over-identified), and this has at least two potential implications for subsequent model comparisons undertaken. First, a saturated model does not permit tests of model (mis) fit. Second, and beyond the present scope since our purpose was to test the prescribed model, a more parsimonious model may have been established at baseline for comparison. The present findings, with several null pathways, suggest a more parsimonious model may be possible. We encourage future research, with a new sample, to confirm this possibility. Regardless of these limitations, the current study is of great significance as it is the first study of objectification that comprised a large sample of Asian and Caucasian participants and took for the first time level of WCI into consideration.

### Implications

Although the present findings do not establish causality, they do establish one precondition (covariation between variables in a proposed causal chain). Insofar as future research establishes through experimental designs that these relationships are causal, there are potential implications. For instance, preventive strategies, regardless of the ethnic background and level of WCI, would benefit from identifying women evidencing high thin-ideal internalization and body surveillance and design prevention efforts targeted to these objectification processes. Such programs might involve psychoeducation of realistic body shapes and sizes, the negative impact of consuming media that objectify women and increasing awareness of the pervasiveness of sexual objectification of women and its implications. Interventions would also benefit from emphasizing individuals’ internal qualities and to provide embodied experiences. Cognitive dissonance-based prevention programs already do this by including behavioral activities whereby participants speak or write positively about their bodies, including their bodies’ physical, emotional, intellectual, and social qualities (Becker et al., 2013).

Furthermore, our findings indicated that the development of eating pathology might differ within the broader context of the objectification model across women of varying levels of WCI. For example, for Asian women who identify more with the western culture, overvaluation of their appearance is more likely to result in maladaptive eating behaviors and attitudes, compared to less-WCI Asian women. Therefore, for the former population, it may be important to focus on distorted cognitions related to the magnified importance of appearances in the development of preventative strategies, early interventions or treatment. As such, it may be relevant to consider levels of stressors related to WCI during assessment.

## Conclusion

To conclude, our study highlights that objectification theory provides a suitable framework to explore the development of eating pathology in Asian women and that the level of WCI does play a role in influencing both state and trait variables within the model. Overall, our findings showed that the Caucasian/High-WCI Asian sample presented with more significant pathways within our revised model than the low-WCI Asian group. Our findings might therefore indicate that the low-WCI Asian women might have protective factors, which might prevent them from the negative psychological consequences of self-objectification. Future research in culturally diverse samples would benefit from conducting more experimental and longitudinal studies to evaluate changes of our revised objectification theory constructs and to clarify the direction of causality in the posited relations in the objectification framework.

## Author Contributions

CT, IK, SL, RU, and MF-T drafted the manuscript and conceptualized the aims and hypotheses. MF-T conducted the analyses. IK, VY, CT, and TP set up data collection. All authors provided feedback on different versions of the manuscripts. All authors read and approved the final manuscript and are accountable for all aspects of the work in ensuring that questions related to the accuracy of any part of the work are appropriately investigated.

## Conflict of Interest Statement

The authors declare that the research was conducted in the absence of any commercial or financial relationships that could be construed as a potential conflict of interest.
